# TransAAP: an automated annotation pipeline for membrane transporter prediction in bacterial genomes

**DOI:** 10.1099/mgen.0.000927

**Published:** 2023-01-18

**Authors:** Liam D. H. Elbourne, Brendan Wilson-Mortier, Qinghu Ren, Karl A. Hassan, Sasha G. Tetu, Ian T. Paulsen

**Affiliations:** ^1^​ School of Natural Sciences, Macquarie University, Sydney, Australia; ^2^​ Biomolecular Discovery Research Centre, Macquarie University, Sydney, Australia; ^3^​ ARC Centre of Excellence in Synthetic Biology, Macquarie University, Sydney, Australia; ^4^​ Memorial Sloan Kettering Cancer Center, New York, USA; ^5^​ School of Environmental and Life Sciences, Newcastle University, Newcastle, Australia

**Keywords:** annotation, bioinformatics, genomics, membrane transporters, web server

## Abstract

Membrane transporters are a large group of proteins that span cell membranes and contribute to critical cell processes, including delivery of essential nutrients, ejection of waste products, and assisting the cell in sensing environmental conditions. Obtaining an accurate and specific annotation of the transporter proteins encoded by a micro-organism can provide details of its likely nutritional preferences and environmental niche(s), and identify novel transporters that could be utilized in small molecule production in industrial biotechnology. The Transporter Automated Annotation Pipeline (TransAAP) (http://www.membranetransport.org/transportDB2/TransAAP_login.html) is a fully automated web service for the prediction and annotation of membrane transport proteins in an organism from its genome sequence, by using comparisons with both curated databases such as the TCDB (Transporter Classification Database) and TDB, as well as selected Pfams and TIGRFAMs of transporter families and other methodologies. TransAAP was used to annotate transporter genes in the prokaryotic genomes in the National Center for Biotechnology Information (NCBI) RefSeq; these are presented in the transporter database TransportDB (http://www.membranetransport.org) website, which has a suite of data visualization and analysis tools. Creation and maintenance of a bioinformatic database specific for transporters in all genomic datasets is essential for microbiology research groups and the general research/biotechnology community to obtain a detailed picture of membrane transporter systems in various environments, as well as comprehensive information on specific membrane transport proteins.

## Significance as a BioResource to the community

Annotation of membrane transporters is a vital step in genome feature elucidation and important to cell phenotype prediction and study. Here, we describe the Transporter Automated Annotation Pipeline (TransAAP), an online resource for annotating membrane transporters in sequenced genomes. This is a resource of broad general interest, aiding fields of study including genomics, metagenomics, metabolic engineering, synthetic biology and systems biology, as well membrane transport. The resource has served the research community for over 15 years, contributing to studies in a wide array of microbial prokaryotes, eukaryotes and, more recently, microbiomes, as evidenced by the large number of publications in discipline-specific, reviews and high-impact journals citing this resource.

## Data Summary

TransAAP is available at http://www.membranetransport.org/transportDB2/TransAAP_login.html.

## Introduction

Membrane transport systems are vital to every living cellular organism. The advent of membrane translocation systems is hypothesized to have occurred early in the development of cellular life, necessitated by the separation of the replicative machinery of the proto-cell from the external environment by the lipid barrier presented by the cell membrane [1]. From these proto-cells onwards, transport systems were crucial, given that passive diffusion through the lipid bilayer severely limits both chemical uptake and efflux [2]. Subsequently, transporter proteins have evolved to function across a vast variety of roles in the acquisition of organic nutrients, maintenance of ion homeostasis, efflux of toxic and waste compounds, environmental sensing and cell communication, and other important cellular functions [3, 4]. Therefore, they underpin essential roles in life-endowing processes like metabolism, communication and reproduction. Comparative genomic analyses have revealed that between 1 and 20 % of all ORFs in prokaryotic and eukaryotic genomes are predicted to encode membrane transport proteins, underscoring the importance of transporters to the lifestyles of these organisms [5, 6].

The variety of membrane transport systems, differentiated by membrane topology, energy coupling mechanisms, phylogeny and substrate specificities, represents a challenge for systematic classification, a requirement for bioinformatic analyses. The Transporter Classification (TC) system (http://www.tcdb.org/) was developed by the Saier laboratory [7], and represents a systematic approach to the classification of transport systems. In a similar manner to the Enzyme Commission number (EC number) scheme that classifies enzymes according to the chemical reactions they catalyse, the TC system classifies transport proteins according to the mode of transport, energy coupling mechanism, molecular phylogeny and substrate specificity [7–11]. There are four characterized classes of solute transporters in the TC system. *Primary active transporters* couple the transport process to a primary source of energy (ATP hydrolysis), e.g. the MalKGFE maltose transporter from *Escherichia coli* [12, 13]. *Secondary transporters* utilize an ion or solute electrochemical gradient, such as the proton/sodium motive force, to drive the transport process, e.g. *E. coli* LacY lactose permease [14–17]. *Group translocators* transport and phosphorylate their substrates, e.g. the *E. coli* MtlA mannitol phosphotransferase system (PTS) transporter phosphorylates exogenous mannitol using phosphoenolpyruvate (PEP) as the phosphoryl donor and energy source, and releases the phosphate ester, mannitol-1-P, into the cell cytoplasm [18, 19]. *Channels* are energy-independent transporters that transport water, specific types of ions or small hydrophilic molecules down a concentration or electrical gradient with higher rates of transport and lower stereospecificity (e.g. *E. coli* GlpF glycerol channel) [20].

In 1980, the first membrane transporter gene was cloned and sequenced, the *lacY* gene in *E. coli* [21, 22]. Since then, there has been an ever increasing amount of DNA sequencing, particularly shotgun-sequenced full genomes, requiring accurate and evidence-based annotation. Historically, this was achieved by manual comparison of well-known sequences such as that of *lacY* with novel sequences, using various bioinformatic tools such blast on an ad hoc basis. Such methods, used for transporter analysis in earlier studies [8, 23, 24], required intensive personal/manual involvement and manual curation, and are no longer feasible given the rate at which genome sequence data is now generated. This has necessitated the development of automated systems for the annotation of transporters.

We have developed a publicly available resource, the Transporter Automated Annotation Pipeline (TransAAP) (http://www.membranetransport.org/transportDB2/TransAAP_login.html) to enable identification of the membrane transporter complement within whole genomes based on methods developed in the process of manual curating of transporters. Where possible, sequences are classified within the TC system. This pipeline has been available for public use since 2007 via the TransportDB and later TransportDB2 website [25]. It provides an easy to use, web-accessible resource, which has processed over 5500 user requests for membrane transport annotation. Here, we describe TransAAP, its implementation, methodology and its context within the field of membrane transporter research.

## Methods and Results

### Overview

**Fig. 1.**
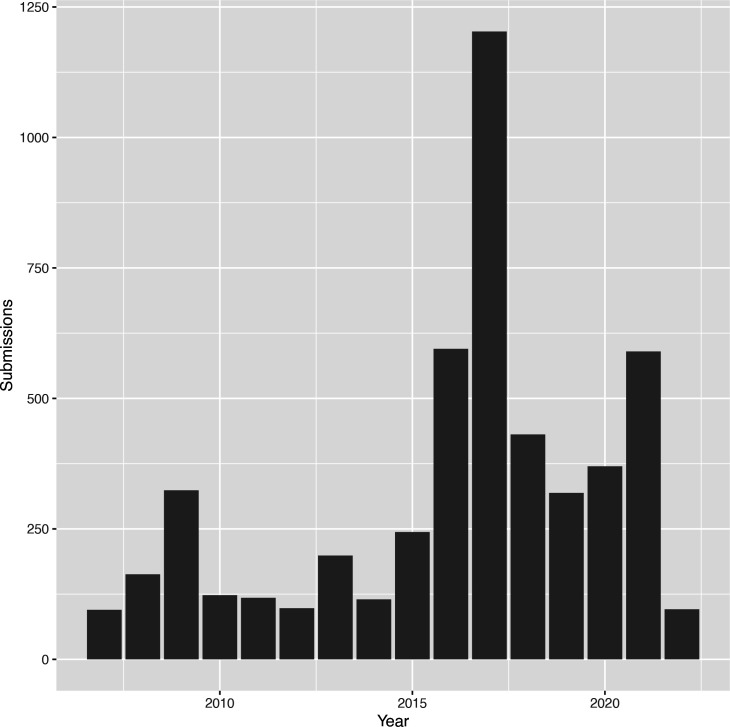
Annual usage of TransAAP since its inception.

The frontend of TransAAP is implemented as a typical LAMP (Linux/Apache/MySQL/PHP-Perl) web application. TransAAP accepts users’ requests for customized transporter annotation via a simple form submission of fasta formatted protein amino acid sequences, National Center for Biotechnology Information (NCBI) taxonomy identifier or FTP link to a fasta file of protein sequences, along with the user details. Subsequently, the amino acid sequences are downloaded to a dedicated Linux cluster that performs the analytical component of TransAAP, elucidated in more detail below. The sequences representing likely transporter candidates are identified and made available through a private version of TransportDB, i.e. can be viewed by the user on the website, and the user is notified by email. The TransAAP annotation process is depicted in a flowchart in Fig. 1.

### Methodology and usage

The methodology/algorithm is based on the manual method initially developed in the Saier laboratory then subsequently the Paulsen laboratory to bioinformatically identify membrane transporters. As such, this depends heavily on ‘expert knowledge’, comparison with existing known transporters and the development of various empirical rules for distinguishing between transporter-like proteins (that may share domains or transmembrane architecture) and actual experimentally characterized transporters.

TransAAP was designed as a web-based transporter annotation tool to make the transporter annotation pipeline available to the public through our TransportDB web portal. TransAAP allows researchers to submit their genomes of interest for transporter annotation via the TransportDB web site after registering as a user. To submit a request for transporter annotation, users fill in Taxonomy ID for genomes deposited in the GenBank database, NCBI genome database name for a NCBI sequenced genome or the fasta format protein sequences/links to FTP protein sequence download site for unfinished genomes. If a GenBank genome has previously been analysed in TransportDB, the user will be sent the link to the relevant page.

All sequences are iteratively run through identity and homology searches on a dedicated Linux cluster. To maximize the possibility of identifying all potential candidate transporter genes, we employ four different kinds of bioinformatic searches with a query sequence. The first is the NCBIs blastp [26] search against our internal manually curated transporter database containing over 100 000 individually curated transporters [5, 25, 27], the TCDB (Transporter Classification Database) [11] and NCBI’s non-redundant (NR) protein database [28]. Secondly, the hmmer3 utility, hmmscan (http://hmmer.org) [29], is used to conduct a profile HMM search against manually curated transporter Pfams (http://xfam.org) [30] and TIGRFAMs (https://www.ncbi.nlm.nih.gov/genome/annotation_prok/evidence/) [31, 32]. Thirdly, also from the NCBI suite of software, rpsblast [26] is used to search against the NCBI COGs (clusters of orthologous groups) database (http://www.ncbi.nlm.nih.gov/COG/) [33]. Finally, a prediction of transmembrane segments is conducted by tmhmm2.0c (https://services.healthtech.dtu.dk/service.php?TMHMM) [34]. These tools are routinely updated, for example, currently we are evaluating the use of DeepTMHMM [35] to supplement tmhmm2, as it can detect a greater range of transporter protein sequence topology. The database searches are mutually inclusive, which means any positive hits above the thresholds set for any of the searches will be aggregated as potential transporters at this stage of the pipeline and stored in a MySQL database. Subsequently, this set is parsed to remove false positives by application of a negative decision tree utilizing heuristics empirically developed to positively identify both transporters and proteins that share features/similarities with characterized transporters, such as ATP-binding domains. This is discussed in more detail below.

### Negative decision tree process: an example

The ABC superfamily of transporters is an ancient, diverse and ubiquitous group of transporters, present throughout all kingdoms of life [36]. This superfamily, which performs both uptake and efflux duties, comprises a large number of families and subfamilies. These are defined based primarily on the significant evolutionary and structural variation in the membrane component, as these are highly conserved, comprising a number of motifs and regions that are well characterized [37–39].The Pfam family ABC_tran PF00005 (https://www.ebi.ac.uk/interpro/entry/pfam/PF00005/) identifies the highly conserved ATP-binding cassette, which binds and hydrolyses ATP, thereby coupling transport to ATP hydrolysis in a large number of biological processes. Besides transporter ATP-binding cassettes, PF00005 also identifies a group of ATPases, e.g. excinuclease ATPase (UvrA), chromosome segregation ATPase (Smc), recombinational DNA repair ATPase (RecF) and ATPases involved in chromosome partitioning (Soj, Mrp), as well as a group of CBS-domain-containing transcriptional regulators and signal transduction proteins. To remove these false positives, we set up a series of negative rules whereby all putative ABC transporters, based on an inclusive and promiscuous search with PF00005, that also have positive hits to the COGs representing these non-transporter ATPases and CBS-domain-containing proteins (shown in Table 1) are deleted. Furthermore, a text search of the annotation of blastp matches to the NR database is also conducted, and where the top hits are found to contain keywords shown in Table 1, these entries are also removed. These rules assist in the specific identification of ATP-binding cassette transporters.

**Table 1.** Negative selection rules for ATP-binding cassettes annotation

**rpsblast** searches against COGs		
COG0178	UvrA	Excinuclease ATPase subunit [DNA replication, recombination and repair]
COG1196	Smc	Chromosome segregation ATPases [cell division and chromosome partitioning]
COG1106	COG1106	Predicted ATPases [general function prediction only]
COG1195	RecF	Recombinational DNA repair ATPase (RecF pathway) [DNA replication, recombination and repair]
COG0419	SbcC	ATPase involved in DNA repair [DNA replication, recombination and repair]
COG3910	COG3910	Predicted ATPase [general function prediction only]
COG1192	Soj	ATPases involved in chromosome partitioning [cell division and chromosome partitioning]
COG0489	Mrp	ATPases involved in chromosome partitioning [cell division and chromosome partitioning]
COG2524	–	Predicted transcriptional regulator, contains C-terminal CBS domains [transcription]
COG2905	–	Predicted signal-transduction protein containing cAMP-binding and CBS domains [signal transduction mechanisms]
COG4109	–	Predicted transcriptional regulator containing CBS domains [transcription]
COG0517	–	FOG: CBS domain [general function prediction only]
**blastp** **searches against NR database**		
cAMP-binding		
CBS domain		
Chromosome partition		
Cytochrome *c* assembly		
Cytochrome *c* biogenesis		
Excinuclease		
RNase L inhibitor		
UvrABC		

The submission database is polled every 24 h and new submissions are automatically processed via the TransAAP pipeline on a dedicated Linux cluster, running the relevant searches and predicting the complete transporter contents, then uploading all the results into the TransportDB MySQL database. Users can check the status of the annotation of their submitted genomes and once complete can access a list of predicted transporters, or individual transporter annotation pages. Users can view annotation, supporting evidence, and curate the annotation on each individual transporter annotation page, or download the result as a tab delimited text file (Fig. 1).

### General outline of the TransAAP: history and usage

**Fig. 2.**
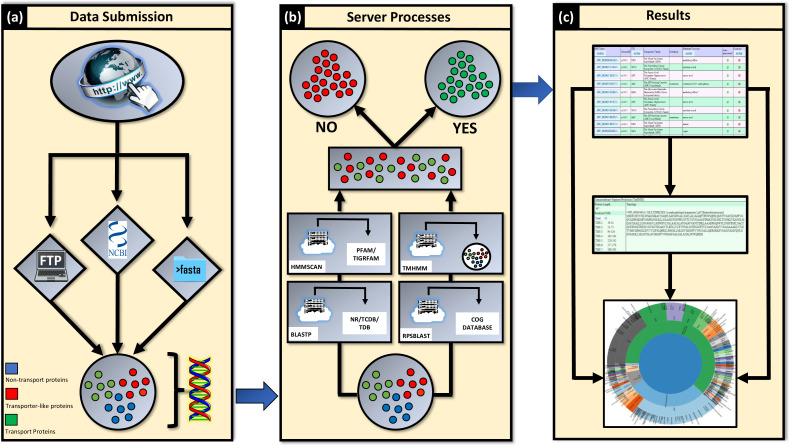
Input to TransportDB (a) can take one of three forms: a fasta file containing the protein amino acid sequence of the genome of interest, the organism’s NCBI taxonomy ID or the NCBI FTP link. Regardless of the chosen method, all genomic protein sequences are extracted for analysis. Following submission, the data is sent to a dedicated local Linux cluster and the protein sequences are searched against various genomic databases with blastp, rpsblastand hmmscan(b, lower) to identify putative ‘transporter-like’ proteins, whilst transmembrane domains are predicted via tmhmm. Sequences that satisfy these parameters are then extracted and filtered by a negative decision tree (b, top), which minimizes the occurrence of false-positives. Upon completion, the user receives an email notification indicating that the job is complete and can retrieve a full list of the predicted transport proteins (c, top). This information includes the full protein sequence, predicted transmembrane domains and predicted substrate specificities (c, middle). Users are also able to use TransportDB to visualize the distribution of the transport proteins within their organism and download the file for further use (c, lower).

Since the inception of TransAAP in mid-2007, we have received over 5500 requests for automated transporter annotation of prokaryotic genomes from a total of 1279 registered users from research groups and commercial entities such as biotechnology companies distributed globally. We also conduct external eukaryotic and metagenomic analyses on an ad hoc or collaborative basis, but these require greater computational resources and manual intervention, so are not totally automated. The annualized usage of TransAAP (Fig. 2) shows persistent use of the pipeline, demonstrating its sustained usefulness to the community.

### Evaluation of TransAAP on genomic transporter annotation

TransAAP is benchmarked against both manually and informatically curated genome sequences and databases such as TCDB [11], which contain both informatically assigned transporters and individual transporters with laboratory determined activity and structural information, and other general annotation pipelines [such as NCBI’s Prokaryotic Genome Annotation Pipeline (pgap)] [32], as well as transporter-specific annotation bioinformatic tools (examples of these are discussed below). The manual annotation utilizes the same set of search results, but involves expert scrutiny of genomic context information and each type of evidence to verify the transporter complement. We also compare our results to projects involving specific organisms, such as the highly manually curated *E. coli* mg1655 in the EcoCyc database [40]. In this instance, the curation is based on literature available on functional assays of proteins in the mg1655 genome, including membrane transporters. In addition, as a research group involved in laboratory-based transporter study, we incorporate information from our own functional studies, such as the discovery of the PACE (proteobacterial antimicrobial compound eﬄux) family of transporters [41]. This benchmarking is carried out with all new iterations of the software, with regressive testing against our own core manually curated database, TDB.

An example of this benchmarking process applied to *Acidiphilium cryptum* JF-5, an *Alphaproteobacteria *, and the archaeal *Thermofilum pendens* Hrk 5, is outlined here and summarized in Table 2. In *Acidiphilium cryptum *, a total of 364 transport proteins were predicted by TransAAP, among which 357 were confirmed by manual annotation. Manual annotation also picked up an additional transport protein. In *T. pendens *, TransAAP identified 221 transport proteins. In total, 212 of them were confirmed by manual annotation. Manual annotation identified three additional transporters. Overall this equates to a low percentage of false positives (2 and 4%, respectively, for the two genomes) and false negatives (0.3 and 1.4%, respectively). TransAAP performed relatively better on *Proteobacteria* than on archaeal species, this may be because we have a much larger number of *Proteobacteria* in our search database, a bias caused by their generally higher representation in sequencing projects.

**Table 2.** Comparison of TransAAP annotation to the manual annotation of a bacterial and an archaeal genome

	* Acidiphilium cryptum* JF-5	* Thermofilum pendens* Hrk 5
Manual annotation	358	215
TransAAP	**364**	**221**
False positves	7	9
False negatives	1	3
Substrate prediction corrections	23	15

The false positives are mainly from the same sources, the CPA3 family (TC no. 2 .A.63), which consists of bacterial multicomponent potassium/sodium ion:proton antiporters. The best characterized of these are the PhaABCDEFG system of *Rhizobium meliloti* that functions in pH adaptation and as a potassium ion efflux system, and the MnhABCDEFG system of *Staphylococcus aureus* that functions as a sodium ion efflux antiporter. Two subunits of this system, PhaA and PhaD, are homologous, respectively, to the ND5 and ND4 subunits of the proton-pumping NADH:ubiquinone oxidoreductase. Therefore, the corresponding protein domain searches will identify other NADH:ubiquinone oxidoreductases and enzymes with a similar functional domain.

The false negatives primarily come from transporter family/subfamilies with less conserved/characterized functional domains. For example, the transmembrane subunits of an ABC transporter can be difficult to identify bioinformatically. Transporters classified in the ABC superfamily consist of three types of subunits: an ATP-binding subunit that is highly conserved, a transmembrane subunit that usually consists of six or more transmembrane segments and, in the case of importers, substrate-binding subunits that are highly specific to their substrate. The transmembrane and substrate-binding subunits are less conserved compared to the ATP-binding subunits, and there are no well-characterized protein domains to represent these subunits. Currently, this is dealt with by including diverse manually curated examples of this superfamily in the internal TDB reference database, as well as using those in the TCDB, to allow for homology transfer based on sequence similarity. Resolving this and similar issues remains an area of active research for the TransAAP development team.

### Substrate prediction

One of the key features of TransAAP is it attempts to predict substrate specificity to the specific substrate level (for example ‘galactose’ as opposed to ‘sugar’), in addition to the general class the substrate belongs too (‘sugars’, ‘amino acids’, etc.) where possible. Substrate prediction is often challenging to perform solely using bioinformatic methodology, however, as many transporters can be difficult to align (manual checking of the alignments may be required in some cases) and phylogenetic tree inference, which is particularly informative [42–44], is currently too computationally intensive for complete automation. TransAAP substrate prediction is based on a combination of homology transfer, comparison with known models in the form of COGs/TIGRFAMs/HMMs, and in-house knowledge encoded as heuristics, that has been empirically established over the lifetime of the pipeline. Based on benchmarking, we estimate a TransAAP substrate prediction accuracy of 93–94 %. When in doubt, substrate specificity predictions are not made, which occurs in a mean of 18 % of transporters over a wide range of species from diverse taxa [45].

Apart from our own benchmarking against known transporters (manually assayed) and comparison with phylogenetic trees of known transporters with experimentally determined substrates, independent studies have confirmed TransAAP’s substrate predictions in the laboratory with functionality studies/assays, particularly with potential drug transporters. For example, Calgin *et al*. [46] confirmed that the expression levels of 15 putative drug efflux transporters predicted by TransportDB were statistically higher in multidrug-resistant strains compared to drug-sensitive strains, which may contribute to the drug-resistant phenotype. Another transcriptomic study conducted by Li *et al*. [47] profiled the expression of putative sugar transporters contained within the genome of the filamentous fungus *Neurospora crassa* based on TransportDB annotations. This resulted in the functional characterization of three novel sugar transporters confirmed by heterologous expression, with transcriptomic data supporting sugar transport for eleven more [47]. Other examples of genes successfully characterized and chosen for analysis based on TransportDB substrate predictions include an ABC-type drug efflux operon in *Bifidobacterium longum* [48], three drug efflux transporters in *Salmonella typhimurium* — one of which conferred resistance to ten antimicrobials when heterologously expressed in a hypersensitive *E. coli* strain [49] — and the confirmation of Zn^2+^ transport in a putative CDF transporter in *Salmonella typhimurium *, and a putative CDF transporter in ' *Aquifex aeolicus* ' [50]. Though the bioinformatic prediction of substrate specificity is not an alternative for the experimental characterization of transport proteins, a high-throughput ligand-binding screening of ABC SBPs (substrate-binding proteins) from *Rhodopseudomonas palustris* identified that TransAAP substrate predictions of glycerol-3-phosphate, phosphate, sulphate and peptide transport were consistent with experimental data, illustrating its usefulness as an adjunct, or precursor, to experimental characterization.

### Usefulness and case studies

TransAAP has provided immense utility to the scientific community. Over the past 15 years, there have been 583 journal publications that have resulted from, or incorporated results from, the use of TransAAP (based on PubMed word searches, January 2022). Additionally, the pipeline has been utilized in another 267 conference proceedings, theses, book chapters and other sources (all sources determined with a text search of Google Scholar using the terms ‘TransportDB’, 'TransAAP' and the URL www.membranetransport.org, and then manual curation of the articles found to determine whether they utilized TransAAP). All these publications encompass studies ranging from analyses of antibiotic resistance through to metabolic modelling across a wide range of different prokaryotes and eukaryotes. We provide here some example case studies, indicating common ways TransAAP analyses have aided biological research.

### Genomic and metagenome analyses

One of the most common uses of transporter analysis has been to contribute to comparative genome analyses, providing critical insight into how different organisms interact with their environment, including what substrates they potentially utilize as nutrient sources and what environmental challenges (including antibiotic or other clinical drug exposure) they may tolerate. For example, Hassan and colleagues applied TransAAP to analyse the drug efflux potential of bacterial strains within the *Bacillus cereus* group, finding that this bacterial lineage devotes substantial protein coding potential (>2.5 % of protein-encoding genes) to the production of drug efflux pumps, more than most other lineages examined to date [51]. This work also identified a number of new putative efflux pump gene targets in this bacterial group, which were then examined experimentally, gaining new insight into their likely functions, many of which were of clinical relevance. TransAAP analyses have also been applied in metagenome-based studies. An example of such is the application of TransAAP by Allen and colleagues to gain substrate utilization insight across a whole community. In this work, Southern California Bight and California Current metagenomes were examined, finding that organisms with small genomes had an enrichment of transporters with substrate specificities for amino acids, iron and cadmium, whereas organisms with larger genomes had a higher percentage of transporters for ammonium and potassium, indicating populations contributing in different ways to nutrient cycling co-existed within these marine environments [52].

### Transporter and transporter substrate prediction for metabolic engineering

Transporter predictions generated by TransAAP have been used to guide microbial metabolic engineering projects. These projects aim to apply micro-organisms in the production of commercially valuable small molecules of interest. Transporters play critical roles in efficient biomolecule production – uptake systems are necessary to import metabolites or product precursors available in the microbial feedstock; the expression of an efflux system specific to the desired metabolic end-product can help to drive optimal molecule production by providing an efficient means of separating it from the cells and reducing end-product inhibition; and conversely, the expression of efflux pumps that recognize precursors to the desired product can reduce substrate flux through the metabolic pathway and, thus, reduce final product yield [53]. Therefore, it is important to identify those transporters within a microbial system that influences productivity. Wang *et al*. recently applied innovative approaches to achieve this in *Saccharomyces cerevisiae* to improve production of *cis*,*cis*-muconic acid, protocatechuic acid and betaxanthins [54]. Data generated by TransAAP was accessed via TransportDB to assist in defining the ‘transportome’ of *Saccharomyces cerevisiae*, which consisted of 411 transporters (388 from TransportDB). The genes encoding all non-essential transporters were targeted for inactivation en masse using a CRISPR-Cas9 system, producing pools of 361 transporter-inactivated mutants in strains engineered for target molecule production [54]. Transcription-factor-based fluorescent protein biosensors were used to detect levels of *cis*,*cis*-muconic acid and protocatechuic acid production in the mutant pools and were screened and enriched using fluorescent-activated cell sorting. Betaxanthin production was visualized directly. The transport activities of the transporters most influential in product generation were validated using targeted mutants and heterologous expression hosts [54].

### The functional transportome and synthetic biology

The first step in developing synthetic cellular machinery is the identification of appropriate enzyme components in natural cells’ functional units. Claus *et al.* [55], interested in the bioproduction of a naturally occurring surfactant produced by *Starmerella bombicola*, utilized TransAAP in conjunction with a number of other bioinformatic tools to assess the transportome of *Starmerella bombicola*. This poorly annotated genome, a draft at the time the study was conducted, had only 343 nucleotide records in NCBI, of which only four were transporters. Of these transporters identified by general annotation, very little information was available on potential substrates. TransAAP was able to annotate 254 transporters and, using a combination of TransAAP, TrSSP and UniProt [56] annotation, substrate utilization was inferred generally on a consensus basis; where differences existed, manual checking suggested that TransAAP had made the correct call. The process identified the specific two ABC transporters responsible for sophorolipid export, one within the sophorolipid biosynthetic gene cluster where its presence was anticipated, but an additional sophorolipid exporter was also found elsewhere in the genome. The inference of these two transporters, and others, in this study, reduced the pool of candidates that needed to be involved in wet lab characterization (i.e. knockout, mutagenesis, etc.), significantly streamlining the process of functional characterization of the cell.

### Other bioinformatic applications for membrane transporter annotation

While the goal of this article is to describe TransAAP, rather than review all approaches to membrane transporter annotation, we present some general discussion of other exemplar tools/databases to provide context to the approach and utility of TransAAP. General genome sequence annotation software, such as those found via web services at NCBI and JGI (Joint Genome Institute) (https://www.ncbi.nlm.nih.gov/genome/annotation_prok/, https://img.jgi.doe.gov/cgi-bin/submit/main.cgi), and/or downloadable software for running locally (NCBI’s pgap software [32], Prokka [57]), all attempt to annotate membrane transporters. These annotation systems will also ascribe potential substrates, generally based on homology transfer from the reference protein (in broad terms based on the match to Pfam/TIGRFAMs/COGs) that matches and homology transfer. Recently, the software that drives the genome annotation provided by NCBI, pgap, has been developed/enhanced in its transporter annotation capability [32], utilizing a similar logical structure to TransAAP with a variety of hand-curated (BlastRules) rules to eliminate false positives and include false negatives. TransAAP attempts a much more specific annotation of the substrate specificity than any of the tools above, and presents the relevant transporter related information (family, subfamily and TCDB number) in an accessible manner.

There are also specific transporter and transporter substrate specificity tools available as web services and/or downloadable software to run locally. Some are components or plugins for packages for metabolic modelling, specifically aimed at providing transporter reactions (such as merlin, which uses TransSyT [58], and Pathway Tools, which uses rast/tip), a task for which TransAAP has also been extensively utilized. Some examples of other transporter tools or databases that exist include TrSSP utilized in the *Starmerella bombicola* analysis example mentioned above [59], an annotation pipeline optimized for eukaryotic organisms that uses homology modelling approaches followed by machine-learning methods to refine predictions, and provides general substrate class, but not specific substrate, and has restrictions on processivity (maximum 2000 fasta sequences). The TCDB [11] is an extensive database resource for transporter research (and is used as a reference database by TransAAP) and classifies exemplar proteins into families (analogous to the Enzyme Commission system). It does not provide an automated membrane transporter annotation service, but does provide access to the individual tools that the Saier laboratory uses internally to characterize transporters [60]. A number of software implementations intended to identify membrane transporter sequences and/or substrate specificity, both local and web based, have also been developed that appear to be no longer extant, or are minimally cited (TransATH, TranCEP, TransportTP, scmmtp, LGICdb, ttrbf, etc.).

## Conclusions

TransAAP is an established and well-utilized tool for membrane transporter analysis. Its implementation as a web service, with straightforward job submission process and a Linux cluster backend, makes it easy to use and relatively fast. Data from TransAAP has been used to support genome and metagenome annotation projects and genome-scale metabolic reconstruction projects that require genome-scale data on transporter function. The methodology, underpinned as it is by empirically derived rules based on expertise in the field, provide reliable and consistent results. One of the inherent advantages of this system springs from it being developed in a laboratory that also actively works to experimentally characterize membrane transporter function, allowing for a cross-fertilization of functional, biochemical, structural and informatic processes, e.g. the PACE transporter family discovery [41]. Attempting to annotate substrate utilization is a key feature of the pipeline, and external studies have verified its high level of accuracy for specific transporters. TransAAP has a provided a sustainable service to the research community for over 15 years and continues to be developed on an active basis, with the goal of continued improvement and applicability to biological and biotechnological research in years to come.
